# The Effectiveness of Novel e-Health Applications for the Management of Obesity in Childhood and Adolescence During the COVID-19 Outbreak in Greece

**DOI:** 10.3390/nu17132142

**Published:** 2025-06-27

**Authors:** Eleni Ramouzi, George Paltoglou, Diamanto Koutaki, Maria Manou, Christos Papagiannopoulos, Athanasia Tragomalou, Aikaterini Vourdoumpa, Penio Kassari, Evangelia Charmandari

**Affiliations:** 1 Division of Endocrinology, Metabolism and Diabetes, First Department of Pediatrics, National and Kapodistrian University of Athens Medical School, ‘Aghia Sophia’ Children’s Hospital, 11527 Athens, Greece; eleni_ramouzi@hotmail.gr (E.R.); gpaltoglou@gmail.com (G.P.); mk_madw@hotmail.com (D.K.); mariamanou93@hotmail.com (M.M.); christospapajohn@gmail.com (C.P.); nansymou@hotmail.com (A.T.); katvourdouba@gmail.com (A.V.); peniokassari@gmail.com (P.K.); 2 Division of Endocrinology and Metabolism, Center of Clinical, Experimental Surgery and Translational Research, Biomedical Research Foundation of the Academy of Athens, 11527 Athens, Greece

**Keywords:** e-Health intervention, childhood obesity, COVID-19 pandemic, digital health tools

## Abstract

**Background/Objectives**: The prevalence of childhood obesity has recently increased, particularly during the COVID-19 pandemic, owing to lifestyle changes as a result of public health regulations and guidelines introduced by governments worldwide. The aim of our study was to evaluate the impact of novel e-Health applications in addressing childhood obesity prior to and during the COVID-19 pandemic. **Methods**: The study was conducted as part of the four-year European project BigO (Horizon2020, No.727688). A total of 86 children and adolescents with overweight and obesity (mean age ± standard error of the mean: 11.82 ± 0.25 years; 49 males, 37 females; 31 prepubertal, 55 pubertal) were studied prospectively for 1 year prior to the pandemic (non-COVID-19 group, *n* = 50) and during the pandemic (COVID-19 group, *n* = 36). Based on the body mass index (BMI), subjects were classified as having morbid obesity (*n* = 40, 46,51%) obesity (*n* = 21, 24.42%), overweight (*n* = 22, 25.58%), and normal ΒΜΙ (*n* = 3, 3.49%) according to the International Obesity Task Force cut-off points. The data collection system utilized the BigO technology platform, which connects to a smartphone and smartwatch to objectively record each patient’s diet, sleep, and physical activity. Participants used the BigO system continuously for 4 weeks and wore the smartwatch for specific periods during the week. Subsequently, they entered a personalized, multidisciplinary lifestyle intervention program for 4 months and used the system again for 4 weeks. **Results**: The key finding was a significantly higher improvement rate in BMI category among children and adolescents during the COVID-19 pandemic (58.3%) compared to before the pandemic (36%). Both groups showed significant reductions in BMI, BMI z-score, insulin resistance indices (homeostatic model assessment and quantitative insulin sensitivity check index), blood pressure, gamma-glutamyl transferase, and insulin concentrations, alongside increases in high-density lipoprotein cholesterol (*p* < 0.01). Notably, the COVID-19 group experienced a significantly greater reduction in BMI z-score at 12 months compared to the non-COVID-19 group (*p* < 0.05). **Conclusions**: Our results reveal that the COVID-19 group demonstrated better compliance with lifestyle interventions and experienced more significant improvements in cardiometabolic risk factors. This suggests that the innovative e-Health applications were successful in managing childhood obesity despite the challenges caused by the COVID-19 pandemic.

## 1. Introduction

Obesity in childhood and adolescence has become a worldwide public health concern. The World Health Organization (WHO) recognizes childhood obesity as one of the most significant health challenges of the 21st century, with a rapidly increasing incidence in the last decades [1]. Several factors contribute to the increase in the prevalence of childhood obesity, including lifestyle changes and lack of healthy dietary and environmental conditions. Sedentary lifestyle and easy access to processed foods, in association with socioeconomic factors, play a significant role in this trend [2]. Overweight and obesity in childhood and adolescence are associated with a wide range of comorbidities, such as a greater risk of cardiometabolic disorders, orthopedic complications, and mental health problems [3], which often persist into adulthood, leading to reduced quality of life and increased morbidity and mortality [4]. Early identification and intervention are crucial in addressing childhood obesity and requires comprehensive and innovative solutions [5].

According to 2019 Childhood Obesity Surveillance Initiative (COSI) data, 37.5% of children aged 2 to 14 years in Greece had overweight or obesity, while 42% of children aged 7 to 9 years were classified as having overweight or obesity [6]. This high prevalence reflects significant public health challenges influenced by various socioeconomic and environmental factors unique to the country. The COVID-19 pandemic, which emerged in late 2019, had a major impact on global health and daily routines and habits, especially for children and adolescents [7]. Public health measures, such as lockdowns, school closures and restrictions on outdoor activities, led to a reduction in physical activity and an increase in screentime and unhealthy eating habits [8]. In addition to social isolation and increased psychological stress, these restrictions increased the prevalence of obesity among children [9]. Furthermore, as healthcare services were overwhelmed with COVID-19 cases, many medical appointments were postponed or delayed, highlighting the importance of digital and remote healthcare interventions in managing obesity in childhood and adolescence [10,11].

To address the above issues, efforts have been made to develop and apply effective interventions, with e-Health tools proving to be promising for prevention and management of obesity in childhood and adolescence. New digital health technologies, such as mobile applications, telemedicine platforms, and wearable devices, help monitor and manage health problems in real time [12,13]. In the context of childhood obesity, these e-Health innovations allow for continuous monitoring of physical activity, dietary intake, and sleep patterns, helping to prevent weight gain and promote healthier lifestyle habits [14]. More specifically, mobile health applications enable users to monitor important health markers, are easy to use, and help individuals to set goals, receive personalized advice, and find information about a healthy lifestyle [15]. In addition, telemedicine platforms play a significant role in managing obesity. Because they allow healthcare providers to offer remote counseling, they provide support via video or phone calls and remote monitoring of patient progress and provide doctors with the opportunity to adjust their treatment plan, especially when traditional healthcare is not available, as was the case during the COVID-19 pandemic [16,17,18]. Finally, wearable devices, such as smartwatches, can collect and assess valuable data on children’s and adolescents’ health status, such as heart rate, quality of sleep, and physical activity. For children and adolescents, these devices offer motivation and help them develop and maintain a healthy lifestyle [19].

The urgency of addressing childhood obesity in Greece is further amplified by the COVID-19 pandemic. The aim of our study was to evaluate the effectiveness of novel e-Health applications in addressing childhood obesity before and during the COVID-19 pandemic. It also determined how the COVID-19 pandemic affected participants’ use of e-Health tools and the overall success of these digital health interventions for managing obesity during the pandemic. This is particularly important, given that the pandemic has led to a rapid increase in the use of telemedicine and other digital health tools. Our results show how easily and effectively digital health tools can be adapted in times of crises. Moreover, our study highlights the critical need for developing and evaluating e-Health applications tailored to local needs, which can help manage effective childhood obesity during emergencies and long-term.

## 2. Patients and Methods

### 2.1. Study Design

This study was conducted as part of the “BigO: Big Data against Childhood Obesity” project (http://bigoprogram.eu, Horizon 2020, No. 727688), a four-year European Union-funded initiative aimed at developing technological and scientific tools to combat childhood obesity. Coordinated by the Department of Electrical and Computer Engineering at Aristotle University of Thessaloniki, Greece, the project brought together 13 partners from Greece, Sweden, Ireland, Spain, and the Netherlands. Its primary focus was the use of big data to gain insight into the environmental, behavioral, and social determinants of obesity in children and adolescents [20,21,22]. Our study was carried out at the Center for the Prevention and Management of Overweight and Obesity in Childhood and Adolescence, Division of Pediatric Endocrinology, Metabolism, and Diabetes, First Department of Pediatrics, National and Kapodistrian University of Athens Medical School, ‘Aghia Sophia’ Children’s Hospital. This study adhered to the Declaration of Helsinki and was approved by the Committee on the Ethics of Human Research of ‘Aghia Sophia’ Children’s Hospital (Approval Number: EB-PASCH-MoM: 29 November 2017, Re: 26660-16 November 2017). Written informed consent was given by all parents or legal guardians, and verbal assent was provided by all participants. All subjects and their parents/guardians were fully informed about the study’s purpose and procedures prior to enrollment.

### 2.2. Study Population

A total of 86 children and adolescents (49 boys; 37 girls) aged 9–18 years (mean age ± SE: 11.82 ± 0.25 years) with overweight or obesity, according to the International Obesity Task Force (IOTF) cut off points, were studied prospectively for 1 year. The participants were divided into two groups based on the timing of their enrollment relative to the COVID-19 pandemic: the non-COVID-19 group included 50 participants followed-up and treated in the year prior to the pandemic, while the COVID-19 group consisted of 36 participants followed-up and treated during the pandemic. Participants showed perfect attendance at follow-up and were regularly evaluated by a multidisciplinary team, which included a Pediatrician, Pediatric Endocrinologist, Dietitian, Fitness Trainer, and a Clinical Psychologist. Each participant followed a personalized lifestyle intervention program that offered tailored guidance on healthy eating, quality sleep, and regular physical activity for both the children and their families [13,22].

### 2.3. Methods

#### 2.3.1. Anthropometric Parameters

A trained observer conducted a comprehensive medical history assessment and clinical examination. Participants’ body weight was measured in light clothing and without shoes using a scale (Seca GmbH & Co. KG, Hamburg, Germany). Their standing height was also measured without shoes using a Harpenden stadiometer (Holtain Limited, Crymych, Dyfed, UK). Body mass index (BMI) was calculated by dividing body weight (in kg) by height (in m) squared and was expressed in kg/m^2^. Blood pressure measurements, including systolic (SBP) and diastolic (DBP) blood pressure, were obtained using a sphygmomanometer with an age-appropriate cuff (Comfort 20/40, Visomat, Parapharm, Metamorphosi, Attiki, Greece).

#### 2.3.2. Data Collection System

The data collection system utilized the BigO technology platform, which collects behavioral and environmental data from children and adolescents using mobile applications and smartwatches. This innovative system allowed the objective documentation of various health metrics. Individuals recorded their dietary intake through the application, providing information on their nutritional habits [20]. They were asked to capture images of the food items they consumed and food commercials and to wear the smartwatch during specific periods throughout the week. The smartwatch assessed sleep patterns, measuring both the duration and quality of sleep, including the number of night-time disturbances, overall sleep duration, and efficiency of rest. Furthermore, it monitored physical activity levels, offering insights into the participants’ exercise routine, including steps taken, active minutes, and heart rate during exercise. Moreover, the BigO platform includes multiple web portals tailored for different users: the Clinical Portal allows healthcare professionals to monitor individual data and provide personalized advice, the School Portal is used to organize educational activities around obesity at school and to coordinate class or group participation in the data collection, the Public Health Portal supports policy makers with population-level data, and the Community Portal shares summaries with the public. While the application does not provide direct personalized feedback to participants, personalization is achieved through clinical guidance and school-based engagement. This structure supports replicability and effective user involvement. This thorough and continuous monitoring allowed for a comprehensive evaluation of the effects of the e-Health intervention on childhood obesity [19].

#### 2.3.3. Intervention Program

All participants arrived at the Endocrine Unit early in the morning on the day of the study. A detailed medical history and clinical examination were performed, including assessment of pubertal stage. Standard anthropometric measurements of weight (Wt), height (Ht), hip circumference (HC), and waist circumference (WC) were obtained by a single qualified healthcare professional. Blood samples for baseline hematologic, biochemical, and endocrinologic investigations were collected at 08:00 h after a 12-h overnight fast. Samples were centrifuged, separated, and stored at −80 °C until assayed [23].

Initially, the participants used the BigO system for four weeks to set initial measurements. This involved recording the eating habits, observing sleep behaviors, and measuring physical activity levels, as previously described. All subjects wore the smartwatch during specific periods of the week, including at least 2 weekdays, 1 weekend day, and 3 nights, to collect detailed data.

Following the initial assessment, participants took part in a comprehensive, personalized, lifestyle intervention program for four months. The intervention involved a thorough evaluation of participants by a pediatric team, including a Pediatrician, Pediatric Endocrinologist, and Dietitian, who assessed daily nutritional habits through a 24 h recall method. Families received guidance on healthier eating practices, emphasizing the reduction of processed foods and the inclusion of vegetables, seasonal fruits, high-fiber grains, low-fat proteins, and beneficial fats, in accordance with USDA recommendations. Personalized meal plans were developed, including three main meals and two snacks [15,23,24].

In addition to dietary recommendations, participants were given advice on sleep quality and duration according to the American Academy guidelines, which recommend 9 to 12 h for children aged 10–12 years and 8 to 10 h for adolescents. Education emphasized the adverse effects of inadequate sleep on metabolism and weight regulation, alongside recommendations to reduce screen time. A certified personal trainer conducted weekly assessments of participants’ physical activities, designing customized exercise programs to encourage regular exercise and promote an active lifestyle. The plan included daily participation in preferred physical activities for 30 to 45 min, such as brisk walking, hiking, jogging, swimming, cycling, or dancing.

After the 4-month lifestyle intervention, participants continued using the BigO system for 4 weeks to document changes in their lifestyle habits and behaviors. All subjects documented what they ate and recorded meals and snacks to check their nutrition. The smartwatch helped them monitor their physical activity, ensuring that they met the recommended exercise each week. In addition, participants assessed their sleep quality, duration, and patterns to identify any improvements or areas needing attention. This extended monitoring period allowed for the collection of valuable data and helped to quantify changes in health behaviors and assess the sustainability of lifestyle modifications over time. Although the BigO system was employed only during two 4-week periods, these intervals were carefully chosen to capture essential behavioral patterns and supported by other monitoring methods. Ultimately, this follow-up phase aimed to reinforce healthy habits and support ongoing adherence to a healthier lifestyle, providing insights into the long-term effectiveness of the e-Health interventions [19,23].

Finally, participants arrived at the Endocrine Unit early in the morning for their annual assessment. A trained observer conducted a thorough clinical examination, which included Tanner staging and standard anthropometric measurements. Subsequently, blood samples for detailed hematologic, biochemical, and endocrinologic investigations were obtained at 08:00 a.m. following a 12 h fast.

#### 2.3.4. Assays

Hematologic analyses were performed using multiple automated analyzers: the ADVIA 2110i (Roche Diagnostics, Mannheim, Germany) for general hematology; the ADVIA 1800 (Siemens Healthcare Diagnostics, Tarrytown, NY, USA) for glucose, total cholesterol, triglycerides (TG), and high-density lipoprotein cholesterol (HDL); and the BN ProSpec nephelometer (Siemens Healthcare Diagnostics, Liederbach, Germany) for apolipoproteins A1 (ApoA1), B (ApoB), and lipoprotein (a) [Lp(a)] via latex particle-enhanced immunonephelometric assays. Hemoglobin A1c (HbA1c) was quantified using reversed-phase cation exchange high-performance liquid chromatography with the HA-8160 analyzer (Arkray, Kyoto, Japan). Insulin concentrations were measured by electrochemiluminescence immunoassay on the Cobas e411 (Roche Diagnostics). Total 25-hydroxyvitamin D (25-OH vitamin D) levels were determined using the Modular Analytics E170 electrochemiluminescence immunoassay system (Roche Diagnostics, Basel, Switzerland).

Insulin resistance was estimated with the homeostasis model assessment (HOMA-IR) using the formula: HOMA-IR = [fasting glucose (mg/dL) × fasting insulin (mU/L)]/405. The quantitative insulin sensitivity check index (QUICKI) was calculated as QUICKI = 1/[log(fasting insulin µU/mL) + log(fasting glucose mg/dL)].

#### 2.3.5. Statistical Analysis

Results are shown as means ± standard error (SE) for continuous variables. All variables assessed at initial and annual assessments were compared by employing repeated-measures analysis of variance test (ANOVA) with the COVID-19 group and non-COVID-19 group as between subjects’ factors. Significant main effects were revealed by Fischer’s (LSD) *post hoc* test. Statistical significance was set at *p* < 0.05.

A priori power analysis was conducted using G*Power 3.1 software to ensure sufficient statistical power for detecting within–between interactions. Specifically, with a predetermined medium effect size (f = 0.25), an alpha level of 0.05, and a total sample size of 86 participants distributed across 2 groups and 2 repeated measurements, the achieved power (1 − β error probability) was 0.9956451. This indicates a high probability of detecting a true effect, should it exist. To control for potential biases, we ensured that both groups were matched for gender and pubertal stage. Specifically, the distribution of participants by sex and pubertal stage was as follows: before COVID, there were 32 males (17 prepubertal, 15 pubertal) and 18 females (6 prepubertal, 12 pubertal); during COVID, there were 18 males (6 prepubertal, 12 pubertal) and 18 females (2 prepubertal, 16 pubertal). Additionally, BMI values were similar between groups. These matching procedures were implemented to minimize confounding effects and enhance the internal validity of the study.

The outcome was considered successful if the respective BMI z-score decreased more than 0.6 at 12 months follow-up or if the respective IOTF category (obese, overweight, normal-BMI) improved by one or more steps. All statistical analyses were performed with the Statistica 8 software (StatSoft, Tulsa, OK, USA).

## 3. Results

### 3.1. Clinical Characteristics of All Subjects

Table 1 presents the clinical characteristics of the study population at initial and annual assessments, including gender, pubertal status, and BMI categories. The study sample consisted of 86 children and adolescents. At baseline, most subjects were pubertal (63.95%), while 36.05% were prepubertal. Regarding BMI categories, 46.51% of the participants were classified as having morbid obesity at initial assessment, which decreased to 27.91% at annual assessment. In addition, following the lifestyle intervention, the percentage of participants with obesity decreased by 6.98% (from 24.42% to 17.44%), while the percentages of participants with overweight and normal BMI showed an increase by 13.95% and 11.63%, respectively.

### 3.2. Reduction in BMI and BMI-z Score, BMI Category Improvement

The program’s success was assessed by changes in BMI categories based on IOTF criteria, which classify children as having obesity, overweight, or normal BMI. Both groups were matched for gender and pubertal staging. BMI was also similar between groups. We observed that 36% of participants in the non-COVID-19 group experienced a shift toward lower BMI percentile categories based on IOTF cutoffs, compared to 58.3% in the COVID-19 group (Table 2). Both groups, those who participated before the COVID-19 pandemic and those who participated during the COVID-19 pandemic, showed a significant reduction in BMI and BMI z-score, with the BMI z-score having decreased by more than 0.7 over the 12-month follow-up period (*p* < 0.001). However, the compliance in the COVID-19 group was notably better, as indicated by the statistically significant reduction in BMI z-score compared to the non-COVID-19 group (*p* < 0.05) (Table 3).

### 3.3. Annual Assessment

At the annual assessment, both groups showed significant changes in various cardiometabolic markers. Specifically, insulin resistance indices (HOMA and QUICKI), SBP, and insulin concentrations decreased significantly, while HDL and total cholesterol concentrations increased in both groups after the 12-month follow-up period. The COVID-19 group also demonstrated a significant reduction in DBP and γGT concentrations over time, compared to the non-COVID-19 group. Moreover, vitamin D concentrations were lower at the initial assessment in the COVID-19 group, while no significant difference was noted between groups at the annual assessment (Table 3).

## 4. Discussion

In our study, we assessed the effectiveness of novel e-Health applications in the management of childhood and adolescent obesity prior to and during the COVID-19 outbreak in Greece. The results demonstrate significant improvements in weight management and cardiometabolic indices in both groups, with the COVID-19 group showing notably better results, including significant reductions in BMI z-score, insulin resistance, blood pressure, and γGT concentrations. These findings suggest that the COVID-19 pandemic may have positively influenced adherence to the proposed interventions and underscore the potential of digital health interventions not only in traditional healthcare but also in periods of healthcare crises, such as the COVID-19 pandemic.

Both groups showed significant reductions in BMI and BMI z-score over the 12-month follow-up period, with the BMI z-score having decreased by more than 0.7 in both the COVID-19 and the non-COVID-19 group. This level of reduction indicates a notable improvement in weight management, which is a key factor in addressing obesity in childhood and adolescence. However, the COVID-19 group demonstrated significantly better compliance, as evidenced by a greater decrease in BMI z-score compared to the non-COVID-19 group (*p* = 0.05). This difference indicates that the lockdown measures of the pandemic, which restricted access to traditional face-to-face healthcare services, may have encouraged greater reliance on e-Health applications for monitoring and guidance. This finding is consistent with other studies that have reported increased engagement with digital health tools during the pandemic, as children and their families looked for alternative ways to manage their health without in-person visits to out-patient clinics [25,26,27]. In addition, studies have shown that telemedicine can enhance access to healthcare services, particularly for individuals in rural areas, leading to improved compliance to treatment programs [16,28]. Although telemedicine provides many benefits, its efficiency can be affected by potential barriers, including limited access to the Internet or devices, such as smartphones, tablets, or computers, lack of familiarity with technology, and language diversity [28].

Our study used the IOTF standards to assess the program’s success in changing BMI categories. A shift toward lower BMI percentile categories based on IOTF cutoffs was observed in 58.3% of participants in the COVID-19 group, compared to 36% in the non-COVID-19 group. A potential explanation for the improved outcomes in the COVID-19 group is that family members were spending more time together at home, which led to cooking homemade food, having dinner together more often, and avoiding takeout and prepared foods, since eating outside was not allowed due to restrictions. This family dynamic contributed to healthier eating habits and improved adherence to the recommended interventions. Mobile applications probably enhanced monitoring and goal-setting for managing children’s weight. Similarly, several studies highlight the positive impact of e-Health interventions on weight management among children and adolescents during the COVID-19 pandemic. The French study NutriNet-Santé found changes in dietary habits during the lockdown period, including a higher frequency of family meals and reduced consumption of ready-to-eat meals [29,30], while other studies highlighted the negative effects of lockdown on eating habits, such as an increase in fast food and sugar consumption [31,32]. It is important to recognize that the pandemic’s impact on dietary habits and e-Health applications usage varied, with other factors like socioeconomic status and access to healthcare services potentially influencing these outcomes.

Our study employed a combination of baseline assessment using the BigO system (for 4 weeks) followed by a 4-month lifestyle intervention program and an extended 4-week monitoring phase to evaluate sustainability. This approach emphasizes continuous monitoring and personalized guidance. In the literature, many studies are shorter-term (e.g., 2–4 months) or focus on immediate changes rather than long-term behavior sustainability. Moreover, while other studies mainly used self-reported information or telehealth visits that focused on weight, diet, and exercise, our study used objective data collected through wearable devices and mobile apps with the BigO system. This allowed us to track not only physical activity but also sleep habits and how often participants were exposed to unhealthy food environments using location data [19,33,34,35]. These distinctions help explain some variations in results and underscore the need for standardized protocols in future research.

Furthermore, we observed significant changes in various cardiometabolic parameters in both groups, such as reductions in insulin resistance (HOMA and QUICKI), systolic blood pressure, and insulin concentrations, along with increases in HDL cholesterol concentration. These changes decrease the risk of developing long-term cardiometabolic disorders associated with childhood obesity. The COVID-19 group showed also significant reduction in diastolic blood pressure and γGT concentrations compared to the non-COVID-19 group. The decrease in γGT is particularly relevant given that obesity is associated with fatty liver disease and other hepatic complications [11,24]. Studies have shown that telemedicine can improve health outcomes for subjects with type 2 diabetes and hypertension, including reductions in glucose concentrations and blood pressure [13]. Moreover, mobile-based health interventions designed to promote physical activity and healthy lifestyle have shown a significant positive impact on cardiometabolic risk factors in individuals with metabolic syndrome [34]. Our study was conducted during the COVID-19 pandemic, distinguishing it from other studies that primarily discuss improvements in cardiometabolic factors related to childhood obesity or chronic diseases. While previous studies highlight the effectiveness of various interventions, this study specifically examines weight management strategies during the COVID-19 pandemic and concurs with previous studies that indicate positive outcomes in cardiometabolic risk factors among children and adolescents with obesity during e-Health interventions [15]. However, further research studies are needed to compare these interventions with traditional care to strengthen the case of incorporating this type of technologies into healthcare systems.

Interestingly, in our study we found that the vitamin D concentration was lower at initial assessment in the COVID-19 group, most likely due to the lockdown regulations during the pandemic, which reduced outdoor activity and sun exposure [36]. At the annual assessment, no significant differences in vitamin D concentrations were observed between the study groups, indicating that the intervention may have contributed to an improvement in healthy lifestyle behaviors, such as dietary modifications or supplementation. Previous studies demonstrated that higher intake of vitamin D was related to a reduced risk of severe COVID-19 in these populations [37]. This observation supports our hypothesis that the intervention most likely contributed to the improved vitamin D concentration. Further studies are needed to evaluate the effect of intervention on vitamin D status in children and adolescents with overweight and obesity.

One of the major strengths of our study is that it focused on the e-Health interventions during a critical period, the COVID-19 pandemic. The study benefits from comparing two different groups: individuals who received care prior to the pandemic and those who received care during the pandemic. Moreover, we were able to assess improvements in a variety of cardiometabolic parameters over the course of a 12-month follow-up. However, the study has some limitations. The key limitation is the selection bias due to non-randomization. Secondly, several factors associated with the pandemic, such as psychological stress and lifestyle modifications, may have influenced the outcomes observed between the two study groups. Furthermore, only patients with good attendance during follow-up were included in the final assessment, and self-reported adherence was evaluated by the attending physician at each visit. Although the overall effectiveness of the intervention is presented, no separate validated tools (e.g., questionnaires) were used to objectively measure compliance. Finally, the sample size may not be sufficient to allow definitive conclusions regarding the long-term effects of e-Health applications to address obesity in childhood and adolescence. Future research studies should include larger sample sizes of randomized controlled trials to validate our findings and to explore their long-term consequences.

## 5. Conclusions

In conclusion, these innovative e-Health tools proved successful in tackling childhood obesity despite the challenges and lifestyle changes owing to the COVID-19 outbreak. Our results demonstrate greater adherence to lifestyle interventions and improved cardiometabolic risk factors in the COVID-19 group of subjects with overweight and obesity. The greater success observed during the COVID-19 pandemic underscores the potential of digital health tools in promoting better compliance and outcomes, particularly in challenging times. These findings indicate that e-Health applications can be alternative options for traditional medical services, providing strategies for managing childhood obesity in healthcare crises. Future research studies should focus on the effectiveness of these e-Health applications in larger populations.

Based on these findings, healthcare professionals may consider integrating e-Health applications into standard clinical practice to support lifestyle modification in the management of childhood obesity. These tools may be particularly valuable in situations where access to in-person care is limited. Additionally, healthcare systems and policymakers should examine the possibility of wider implementation of such digital tools as part of comprehensive, long-term strategies to address childhood obesity.

## Figures and Tables

**Table 1:** Gender, pubertal status, and BMI category of all subjects at initial and annual assessment.

	Initial Assessment	Annual Assessment
Gender		
Male	50 (58.14%)	
Female	36 (41.86%)	
Pubertal status		
Prepubertal	31 (36.05%)	17 (19.77%)
Pubertal	55 (63.95%)	69 (80.23%)
BMI category		
Morbid obesity	40 (46.51%)	24 (27.91%)
Obesity	21 (24.42%)	15 (17.44%)
Overweight	22 (25.58%)	34 (39.53%)
Normal BMI	3 (3.49%)	13 (15.12%)

Abbreviations: BMI: body mass index; categorical variables are presented as frequencies (percentages).

**Table 2:** BMI z-score and BMI category improvement in all subjects with “good compliance’’.

	BMI z-Score		BMI Category (IOTF)	
	Non-COVID-19	COVID-19	Non-COVID-19	COVID-19
Successful	12 (24%)	19 (52.7%)	18 (36%)	21 (58.3%)
Not successful	38 (76%)	17 (47.3%)	32 (64%)	15 (41.7%)
Total	50	36	50	36

Abbreviations: BMI: body mass index. All measured variables were compared by employing repeated-measures ANOVA. Significant main effects were revealed by LSD post hoc test.

**Table 3:** BMI, BMI z-score and cardiometabolic parameters in both groups at initial and annual assessment.

	Initial Assessment			Annual Assessment			
	Non-COVID-19	COVID-19	*p*-Value	Non-COVID-19	COVID-19	*p*-Value	*p* Between Timepoints
BMI z-score	2.47 ± 0.2	2.35 ± 0.18	NS	2.21 ± 0.19	1.65 ± 0.15	0.05	0.01/0.01
BMI (kg/m^2^)	28.71 ± 0.82	28.34 ± 0.69	NS	27.76 ± 0.74	25.93 ± 0.57	NS	0.01/0.01
HOMA	4.67 ± 0.4	5.12 ± 0.53	NS	6.00 ± 0.64	3.89 ± 0.32	0.01	0.05/0.05
QUICKI	0.14 ± 0.001	0.135 ± 0.002	NS	0.133 ± 0.002	0.139 ± 0.002	0.01	0.05/0.05
HDL (mg/dL)	46.67 ± 1.37	47.86 ± 2.03	NS	50.49 ± 1.56	51.56 ± 1.71	NS	0.01/0.01
γGT (U/L)	14.35 ± 0.85	15.31 ± 2.62	NS	15.59 ± 1.014	13.17 ± 1.25	0.2	NS/0.05
SBP (mmHg)	113.5 ± 2.087	110.31 ± 2.15	NS	110.29 ± 1.74	104.96 ± 1.76	0.01	NS/0.01
DBP (mmHg)	73.72 ± 2.34	70.65 ± 1.33	NS	70.13 ± 1.58	65.91 ± 1.14	NS	NS/0.05
Insulin (μUI/mL)	20.88 ± 1.74	22.65 ± 2.31	NS	25.22 ± 2.31	17.5 ± 1.4	0.01	NS/0.05
25-OH vitamin D (ng/mL)	23.23 ± 2.07	22.30 ± 2.36	0.05	21.63 ± 2.92	23.64 ± 1.02	NS	NS/NS

Abbreviations: BMI, body mass index; DBP, diastolic blood pressure; γ-GT, serum γ-glutamyltransferase; HDL, high-density lipoprotein; 25-OH vitamin D, total 25-hydroxyvitamin D; SBP, systolic blood pressure. All measured variables were compared by employing repeated-measures ANOVA. Significant main effects were revealed by LSD post hoc test. Statistical significance was set at (*p* < 0.05, rounded to 0.05 in Table), while strong significance (*p* < 0.01, rounded to 0.01 in Table) is also noted. NS: non-significant (*p* > 0.05) difference.

## Data Availability

The data from this study can be obtained by contacting the corresponding author. They are not available for public access due to privacy concerns.
